# Percepções, expectativas e barreiras à cirurgia robótica de artroplastia total do joelho: Estudo transversal com 218 pacientes

**DOI:** 10.1055/s-0045-1812466

**Published:** 2025-11-21

**Authors:** Eduardo Frois Temponi, Álvaro Coura Castro, Renato Monteiro de Castro Junior, Rômullo Vinícius Dutra Menezes, Lúcio Honório de Carvalho Júnior

**Affiliations:** 1 Hospital Madre Teresa, Belo Horizonte, MG, Brasil; 2 Pontifícia Universidade Católica de Minas Gerais, Belo Horizonte, MG, Brasil; 3 Departamento do Aparelho Locomotor, Faculdade de Medicina, Universidade Federal de Minas Gerais, Belo Horizonte, MG, Brasil

**Keywords:** artroplastia do joelho, economia da saúde, procedimentos cirúrgicos robóticos, arthroplasty, replacement, knee, health economic, robotic surgical procedures

## Abstract

**Objetivo:**

Avaliar as percepções e expectativas dos pacientes brasileiros sobre a cirurgia ortopédica robótica do joelho, abordando seu conhecimento prévio, receios relacionados à técnica, impacto econômico e aceitação da tecnologia.

**Métodos:**

Estudo transversal observacional quantitativo, utilizando questionário estruturado aplicado a 218 pacientes ortopédicos com indicação de artroplastia do joelho, atendidos em ambulatório especializado. Foram analisadas variáveis sociodemográficas, fontes e nível de informação, expectativas quanto a benefícios e receios relacionados à cirurgia robótica, além do impacto econômico na tomada de decisão. Dados analisados por estatística descritiva e inferencial (teste do Qui-quadrado e regressão logística), com significância determinada por *p* < 0,05.

**Resultados:**

A maioria dos pacientes (75%) possuía conhecimento superficial sobre a cirurgia robótica, sendo as principais fontes médicos assistentes (40%) e internet/redes sociais (35%). A cirurgia robótica foi considerada positiva por 60%, com expectativas relacionadas à maior precisão (70%) e durabilidade do implante (55%). Entretanto, preocupações sobre alto custo (50%) e falta de controle humano (30%) foram frequentes. A disposição para pagamento pessoal pelo procedimento foi baixa (30%), principalmente devido ao alto custo e à ausência de cobertura por planos de saúde.

**Conclusão:**

Os pacientes apresentam interesse elevado pela cirurgia robótica, mas permanecem hesitantes devido a receios financeiros e falta de informação adequada. Estratégias educacionais e modelos de financiamento mais acessíveis podem aumentar significativamente a aceitação e adoção dessa tecnologia no contexto brasileiro.

## Abstract (en)

**Objective:**

To assess the perceptions and expectations of Brazilian patients regarding robotic orthopedic knee surgery, addressing their prior knowledge, technique-related concerns, economic impact, and acceptance of the technology.

**Methods:**

The present quantitative, observational, cross-sectional study employed a structured questionnaire administered to 218 orthopedic patients undergoing knee arthroplasty at a specialized outpatient clinic. The study evaluated sociodemographic variables, information sources and levels, expectations regarding benefits, concerns related to robotic surgery, and the economic impact on decision-making. Data analysis used descriptive and inferential statistics (Chi-squared test and logistic regression), with significance set at *p* < 0.05.

**Results:**

Most patients (75%) had superficial knowledge about robotic surgery. Their chief sources of information were the attending physicians (40%) and the internet/social media (35%). In total, 60% of the patients had a positive attitude about robotic surgery, with expectations regarding greater precision (70%) and implant durability (55%). However, concerns about high costs (50%) and lack of human control (30%) were common. Willingness to pay for the procedure out of pocket was low (30%), mainly due to its high cost and lack of health insurance coverage.

**Conclusion:**

Patients showed high interest in robotic surgery, but they remained hesitant due to economic concerns and the lack of adequate information. Educational strategies and more accessible financing models can significantly increase the acceptance and adoption of this technology in Brazil.

## Introdução

A cirurgia robótica tem se consolidado como uma inovação promissora na ortopedia, particularmente em procedimentos de reconstrução articular, como a artroplastia total do joelho (ATJ). Essa tecnologia oferece maior precisão, reprodutibilidade e personalização no planejamento cirúrgico, com potencial para melhores resultados funcionais e menor taxa de complicações pós-operatórias. 1 2 3

Embora sua adoção esteja em expansão nos cenários internacional e nacional, a literatura ainda é limitada quanto à compreensão das percepções e expectativas dos pacientes em relação a esse tipo de procedimento, sobretudo no período pré-operatório. 4 5 Esses aspectos são relevantes, pois influenciam diretamente a tomada de decisão compartilhada, a adesão ao tratamento e a satisfação com os resultados cirúrgicos. 6

No contexto brasileiro, não há estudos publicados que avaliem de forma sistemática o conhecimento, os receios e as barreiras percebidas por pacientes com indicação de artroplastia quanto ao uso da tecnologia robótica. Considerando que essas percepções são frequentemente moldadas por informações de fontes diversas—como médicos, mídia e redes sociais, nem sempre precisas ou baseadas em evidência, torna-se essencial identificar possíveis lacunas no entendimento do paciente. 7

Este estudo tem como objetivo avaliar o nível de conhecimento, as expectativas e os principais temores de pacientes brasileiros com indicação de ATJ em relação à cirurgia robótica, contribuindo para orientar estratégias de educação e comunicação médico-paciente mais eficazes.

## Materiais e Métodos

Este é um estudo observacional, transversal e analítico, com abordagem quantitativa, realizado por meio da aplicação de um questionário estruturado ( **Anexo 1** ) a pacientes atendidos em um ambulatório de cirurgia do joelho com equipe especializada em cirurgias de substituição articular. O estudo foi realizado entre janeiro e maio de 2025. A população-alvo foram pacientes ortopédicos com indicação de ATJ, atendidos em um centro de referência em cirurgia ortopédica.

O questionário utilizado foi previamente testado e adaptado com base no instrumento desenvolvido por Pagani et al., 6 visando garantir clareza, reprodutibilidade e aplicabilidade no contexto brasileiro.

A amostra foi calculada considerando um universo de 500 cirurgias robóticas anuais, um nível de confiança de 95% e um erro amostral de 5%, resultando em um número mínimo de 218 pacientes. Os critérios de inclusão foram: 1) pacientes ≥ 18 anos com indicação cirúrgica para artroplastia do joelho; e 2) consentimento formal mediante assinatura do Termo de Consentimento Livre e Esclarecido (TCLE). Os critérios de exclusão foram: 1) pacientes com déficit cognitivo ou psiquiátrico grave que impedissem a compreensão do questionário; e 2) pacientes que já realizaram cirurgia robótica do joelho.

Os dados foram tabulados e analisados utilizando o software IBM SPSS Statistics for Windows (IBM Corp.), versão 26.0 e GraphPad Prism 9 (GraphPad Software, Inc.). Variáveis categóricas foram apresentadas como frequências absolutas (%) e variáveis contínuas como médias e desvios padrão (DPs). Para análise inferencial, utilizaram-se o teste do Qui-quadrado (χ ^2^ ) e regressão logística, adotando-se significância estatística de *p* < 0,05.

O estudo foi aprovado pelo Comitê de Ética em Pesquisa sob o protocolo CAAE- 78506523.4.0000.5127.

## Resultados

Os resultados foram organizados em cinco blocos principais para facilitar a interpretação e análise estatística: 1) perfil demográfico; 2) *questões sociais* ; 3) *conhecimento e informação* ; 4) *preferências tecnológicas* ; e 5) *impacto financeiro e barreiras econômicas* .

O estudo revelou que, no *perfil demográfico* , dos 218 participantes, a faixa etária predominante foi de 55 a 70 anos (40%), seguida por 40 a 54 anos (35%) e maiores de 70 anos (25%). A maioria era composta por homens (60%) ante mulheres (40%), com rendas predominantemente baixas a moderadas: até 2 salários mínimos (SMs) para 50% dos participantes, entre 2 e 5 SMs para 35% e acima de 5 SMs para 15% deles ( Tabela 1 ).

**Tabela 1:** Conhecimento e Informação sobre cirurgia robótica

Aspecto	Categoria/Opção	Percentual
**Aceitação da cirurgia**	*Sim, aceitaria sem restrições*	35%
	*Sim, mas com ressalvas*	50%
	*Não aceitaria*	15%
**Principais preocupações**	*Alto custo*	50%
	*Falta de controle humano*	30%
	*Incerteza sobre segurança*	20%
**Conhecimento prévio**	*Sim*	75%
	*Não*	25%
**Fonte de informação**	*Médico assistente*	40%
	*Internet e redes sociais*	35%
	*Outros pacientes/família*	25%
**Nível de compreensão**	*Boa/Excelente*	30%
	*Média*	40%
	*Pobre/Muito Pobre*	30%

Quanto às *questões sociais* , a aceitação da cirurgia robótica mostrou-se alta, mas acompanhada de ressalvas, com 50% dos participantes demonstrando preocupações relacionadas ao alto custo (50%), à falta de controle humano no procedimento (30%) e à segurança (20%) ( Tabela 1 ).

No que se refere ao *conhecimento e informação* , 75% dos participantes relataram ter conhecimento prévio sobre cirurgia robótica, sendo as principais fontes de informação os médicos assistentes (40%) e as redes sociais/internet (35%). Contudo, apenas 30% declararam ter um bom/excelente nível de compreensão do procedimento ( Tabela 1 ).

Já em *preferências tecnológicas* , a percepção dos benefícios da cirurgia robótica foi positiva, sendo destacada pela maior precisão (70%) e maior durabilidade do implante (55%). A cirurgia robótica foi preferida por 60% dos pacientes, enquanto 25% se mantiveram indecisos, evidenciando a necessidade de maior esclarecimento ( Tabela 2 ).

**Tabela 2:** Preferências Tecnológicas na escolha do procedimento cirúrgico

Aspecto	Categoria/Opção	Percentual
**Percepção de benefícios**	*Maior precisão*	70%
	*Recuperação mais rápida*	45%
	*Menos dor no pós-operatório*	35%
	*Maior durabilidade do implante*	55%
**Preferência entre cirurgia robótica e convencional**	*Preferência pela robótica*	60%
	*Indeciso*	25%
	*Preferência pela convencional*	15%

Por fim, em *impacto financeiro e barreiras econômicas* , o alto custo e a falta de cobertura pelos planos de saúde aparecem como os principais fatores limitantes. Apenas 45% dos participantes indicaram disposição para arcar com os custos ( Tabela 3 ).

**Tabela 3:** Impacto financeiro e barreiras econômicas para escolha da cirurgia robótica

Aspecto	Categoria/Opção	Percentual
**Disposição para pagar**	*Pagaria do próprio bolso*	55%
	*Não pagaria*	45%
**Fatores que influenciam a decisão**	*Alto custo sem cobertura do plano de saúde*	55%
	*Falta de certeza sobre os benefícios adicionais*	35%
	*Preferência por alternativas convencionais mais acessíveis*	10%

Adicionalmente, foi investigada a percepção dos pacientes sobre o papel do robô em diferentes etapas da cirurgia de substituição do joelho, como incisão, cortes ósseos, equilíbrio ligamentar, implante e fechamento da incisão. Os resultados foram apresentados em tabela, fornecendo maior clareza e detalhamento sobre as opiniões dos pacientes ( Tabela 4 ).

**Tabela 4:** Distribuição das respostas sobre o papel do robô em cada etapa da cirurgia

Etapa da Cirurgia	Todo o processo	Maior parte do processo	Algumas partes do processo	Poucas partes do processo	Não sei dizer
**Incisão (corte da pele até o osso)**	15%	20%	25%	30%	10%
**Cortes ósseos (preparo do osso para a prótese)**	35%	30%	20%	10%	5%
**Balanço ligamentar (ajuste dos tecidos para equilíbrio da prótese)**	25%	35%	25%	10%	5%
**Implante (fixação da prótese no osso)**	20%	30%	30%	15%	5%
**Fechamento da incisão (sutura da pele e tecidos)**	10%	15%	20%	40%	15%

## Discussão

### Conhecimento e Percepções sobre Cirurgia Robótica em ATJ

Os resultados deste estudo revelam um panorama importante sobre as percepções, expectativas e barreiras relacionadas à cirurgia robótica para ATJ no contexto brasileiro. O conhecimento superficial sobre a técnica, relatado por 75% dos participantes, é semelhante aos achados de Chang et al. 1 e Dandamudi et al., 2 que apontaram limitações no entendimento dos pacientes, mesmo diante do crescente interesse pela tecnologia.

As principais fontes de informação identificadas em nosso estudo (médicos assistentes 40%, redes sociais/internet 35%) também foram observadas por Pagani et al. 6 em uma análise de percepção pública baseada em crowdsourcing, na qual médicos e mídias digitais figuraram entre as principais referências informativas.

### Expectativas e Benefícios Percebidos

A percepção positiva da cirurgia robótica por 60% dos participantes, com expectativas relacionadas à maior precisão (70%) e durabilidade do implante (55%), é coerente com a literatura recente. Estudos demonstraram que a cirurgia robótica é associada a menor variabilidade no alinhamento dos componentes, maior acurácia e melhores desfechos objetivos e subjetivos em comparação à técnica convencional. 7 8 Meta-análises recentes confirmam que a precisão no posicionamento e a redução de outliers são benefícios consistentes da abordagem robótica. 9 10

Embora tais ganhos técnicos sejam promissores, alguns estudos sugerem que os benefícios clínicos em médio prazo, como função ou satisfação do paciente, ainda não apresentam diferenças estatisticamente significativas em relação à cirurgia manual. 11 Ainda assim, há evidências de melhor recuperação funcional precoce e menor tempo de internação em pacientes operados por via robótica. 8

### Preocupações e Barreiras à Adoção

Entre os receios relatados, o alto custo (50%) e a percepção de perda de controle humano (30%) se destacaram. Essas preocupações já foram amplamente documentadas na literatura internacional, 2 6 sendo reconhecidas como os principais obstáculos para a adoção da robótica, especialmente em sistemas de saúde públicos ou com restrições de cobertura.

A ansiedade tecnológica, expressa pelo receio quanto à autonomia do robô, também foi relatada por Khlopas et al. 12 e parece estar relacionada à desinformação sobre o papel colaborativo entre cirurgião e tecnologia. Essa desconfiança, no entanto, tende a diminuir após esclarecimentos mais detalhados, reforçando a importância da educação pré-operatória. 2 13

Nos dados do nosso estudo, os pacientes demonstraram maior confiança na utilização do robô em tarefas técnicas específicas, como os cortes ósseos (35%), e menor aceitação no fechamento da incisão (10%), o que também foi observado por Dandamudi et al. 2

### Impacto Econômico e Acessibilidade

A baixa receptividade à possibilidade de ter de arcar com custos adicionais (45%) confirma o papel da limitação financeira como uma barreira estrutural para a ampliação da cirurgia robótica no Brasil. Tal achado se alinha aos dados de estudos que demonstram que a maioria dos pacientes, especialmente aqueles em faixas de renda mais baixas, é sensível ao impacto financeiro dessa tecnologia. 2 14

Modelos de custo-efetividade, como os propostos por Rajan et al., 14 indicam que a robótica pode ser custo-efetiva em longo prazo, especialmente em centros de alto volume e com menor taxa de complicações e revisões. Porém, a realidade brasileira, com acesso restrito e cobertura limitada pelos planos de saúde, torna sua ampla adoção um desafio.

### Comparação com Outras Tecnologias Ortopédicas Emergentes

A resistência inicial à cirurgia robótica segue um padrão semelhante à introdução de outras tecnologias como navegação computadorizada e implantes personalizados. Estudos mostram que as mesmas barreiras – custo, curva de aprendizado e incerteza quanto a benefícios clínicos – foram enfrentadas durante essas transições. 15 16

### Implicações para a Educação e para as Políticas de Saúde

Os resultados deste estudo revelam que a aceitação da cirurgia robótica pelos pacientes ainda é limitada por barreiras informacionais e econômicas. O desconhecimento sobre o funcionamento da tecnologia, somado a dúvidas quanto à sua segurança, reforça a necessidade de estratégias educativas que promovam informação clara, acessível e baseada em evidências, especialmente no momento pré-operatório.

Do ponto de vista institucional, políticas de saúde devem incorporar modelos de financiamento que reduzam o impacto do custo elevado, ampliem a cobertura por convênios e incentivem, progressivamente, a incorporação da cirurgia robótica no sistema público.

Assim, a expansão segura e equitativa da cirurgia robótica no Brasil depende de ações integradas que aliem educação em saúde à reformulação de políticas de acesso, promovendo maior autonomia na tomada de decisão e equidade na oferta da tecnologia. 17 18

### Limitações do Estudo e Direções Futuras

Embora este estudo forneça informações relevantes sobre as percepções dos pacientes brasileiros em relação à cirurgia robótica do joelho, algumas limitações devem ser consideradas. Em primeiro lugar, a amostra foi composta por pacientes de um único centro especializado, o que pode limitar a generalização dos achados para outras regiões do Brasil com diferentes perfis socioeconômicos e culturais. Além disso, por se tratar de um estudo transversal, não é possível avaliar a evolução das percepções ao longo do tempo ou o impacto de intervenções educativas formais.

Outra limitação diz respeito à natureza autorrelatada do questionário, que pode estar sujeita a viés de memória ou desejabilidade social. Ademais, a ausência de comparação direta com pacientes submetidos à cirurgia convencional impede uma análise mais aprofundada sobre diferenças reais de expectativa e compreensão entre os grupos.

Futuras pesquisas devem considerar o delineamento longitudinal, avaliando as percepções dos pacientes antes e após sessões educativas ou mesmo após a realização da cirurgia. Estudos multicêntricos, com maior representatividade geográfica e demográfica, também são recomendados para ampliar a validade externa dos resultados. Além disso, a inclusão de análises qualitativas pode fornecer insights mais profundos sobre motivações, receios e fatores subjetivos que influenciam a decisão dos pacientes.

## Conclusão

Este estudo evidenciou que, embora a cirurgia robótica em ATJ seja percebida positivamente por grande parte dos pacientes, ainda existem lacunas significativas de conhecimento e barreiras econômicas que limitam sua ampla aceitação. A maioria dos participantes demonstrou interesse na tecnologia, reconhecendo benefícios potenciais tais como maior precisão e durabilidade dos implantes. No entanto, preocupações com o alto custo, ausência de cobertura por planos de saúde e a percepção de perda do controle humano no procedimento foram fatores decisivos para a hesitação. Esses achados reforçam a necessidade de estratégias educativas direcionadas, ampliação do acesso à informação qualificada e desenvolvimento de modelos de financiamento que tornem a tecnologia mais acessível. A adoção bem-sucedida da cirurgia robótica no contexto brasileiro dependerá não apenas de sua eficácia clínica, mas também da superação dessas barreiras informacionais e socioeconômicas.
